# Simulation Method for Blockchain Systems with a Public Chain

**DOI:** 10.3390/s22249750

**Published:** 2022-12-12

**Authors:** Yang Liu, Yuxi Zhang, Zhiyuan Lin, Zhaoguo Wang, Xuan Wang

**Affiliations:** 1 School of Computer Science and Technology, Harbin Institute of Technology, Shenzhen 518055, China; 2 Research Center for Cyberspace Security, Peng Cheng Laboratory, Shenzhen 518055, China; 3 Key Laboratory of Novel Security Intelligence Technologies, Shenzhen 518055, China

**Keywords:** blockchain, simulation platform, public chain, smart contract

## Abstract

The potential security problems of blockchain technology are constantly restricting the development process of related industrial applications. The cost of deploying a blockchain system in a real environment to conduct research on security issues is relatively high, and the related security analysis and verification are also destructive and irreproducible. Therefore, based on the idea of layered design, this paper proposes a blockchain system simulation platform. The blockchain system is divided into four layers in the simulation platform: the consensus layer, network layer, contract layer, and storage layer. In the consensus layer, the problem of computing resource waste is solved. In the network layer, a peer-to-peer network topology simulation is implemented. In the storage layer, the problem of redundant storage is solved. In the contract layer, the contract replay speed is accelerated. Finally, a prototype of an efficient blockchain simulation system is implemented based on the above methods.

## 1. Introduction

Blockchain is a special data structure that maintains block states and transaction records. Each block contains multiple transactions, so a blockchain can be viewed as a log of ordered transactions. A blockchain is essentially a distributed ledger that is maintained by a series of nodes that are not fully trusted by each other [1]. All nodes in the blockchain network maintain the same block sequence and reach a consensus by jointly following the agreed rules. Transactions are recorded in the blockchain, where the content is shared by other nodes in the network and cannot be tampered with. This special data structure and operation mechanism cause blockchain technology to have the characteristics of decentralization, anonymity, tamper-proof, security, and stability [2].

With its unique trust-building mechanism, blockchain technology is changing the application scenarios and operating rules of many industries [3]. With the continuous application of blockchain technology in all walks of life, its technical limitations and security problems, such as its consensus mechanism, private key management, and smart contracts, have become become increasingly prominent, and application security events based on blockchain platforms are also endlessly emerging.

The blockchain system itself faces a variety of potential security risks, such as eclipse attacks [4], witch attacks, and DDoS attacks [5,6]. Smart contracts themselves can also be subject to re-entrance attacks [7] due to code flaws. There are similar Ponzi schemes [8], blockchain network data analyses [9], and other ways to threaten the security of ordinary users. For example, on 10 August 2021, Poly Network, a cross-chain interoperability protocol, was hacked to steal 610 million dollars worth of tokens.

The security problems of blockchain technology have seriously restricted the development process of relevant industrial applications [10]. Therefore, more and more people have carried out research in such fields as transaction record analysis [11], smart contract vulnerability detection [12], smart contract virtual machine security enhancement [13], consensus algorithm improvement [14,15], and so on. However, due to the complexity of the blockchain system and the particularity of some security issues, the cost of deploying the blockchain system in a real environment to carry out the above security research is relatively high. In addition, the relevant security analysis and verification are also destructive and unrepeatable, which leads to the difficulty of centralized collection and analysis of relevant analysis and verification data.

All of these constraints in reality hinder the research on relevant safety protection technology [16]. Therefore, in order to deal with the various security risks of the current blockchain system and meet the urgent need for security verification of various algorithms, it is necessary to implement a high-fidelity blockchain simulation system.

The building of a blockchain simulation platform can be used to test the performance characteristics of a variety of different algorithms, evaluate the data processing capabilities and overall performance of different blockchain platforms, and solve the problem of unified evaluation of algorithms due to differences in algorithms’ principles and application scenarios. This plays a key role in in-depth research on key blockchain technologies, helping developers analyze bottlenecks, understand limitations, make efficient decisions, and optimize private blockchain platforms.

At present, according to the openness of the blockchain system and different application scenarios, blockchains can be divided into public chains, alliance chains, and private chains. Although various blockchain systems are different in their specific implementations, their overall architectures have some commonalities. The simulation method designed in this paper will be carried out according to the commonalities of various blockchain systems. On the one hand, a typical blockchain system is simulated to highly reproduce the real blockchain operating environment, meet the needs of relevant security analyses, provide important support for the verification of specific security technologies, and ensure the authenticity of the simulation environment. On the other hand, a variety of performance optimization schemes will be designed and implemented for the specific application scenario of a blockchain simulation to meet the throughput requirements in the simulation scenario and ensure the high efficiency of the simulation environment. Based on the direct simulation method, this paper studies relevant simulation technologies in the consensus layer, network layer, storage layer, and contract layer. The main contributions of this paper are as follows:

1. For the consensus layer, we propose a proof-of-work mechanism simulation technology based on a probability density function. This method can simulate the running state of a blockchain network under any difficulty and any hash rate and realize the high-fidelity simulation of the block generation time of the proof-of-work algorithm.
2. We implement a network layer simulation. In the network layer, we simulate the network protocol and communication link. In the network protocol simulation, this paper implements a node discovery protocol and data communication protocol. The simulation of the underlying communication link is realized with a pipe technology.
3. For the storage layer, we propose a storage optimization mechanism based on shared storage and sequential reading and writing. This method can improve the query performance and solve the problem of redundant data storage.
4. For the contract layer, we propose a parallel replay technology of smart contracts in a simulation environment. This method can solve the problem of wasting computing resources caused by repeated replays of historical blocks, and it can also make full use of the processor performance to speed up block replays.

Section 2 introduces the related work. Section 3 introduces the system architecture of the simulation platform, including the logical level used by the simulation platform, as well as the relationship between the simulation network, simulation node, and blockchain protocol. Section 4 describes the consensus layer to introduce the proof-of-work simulation technology based on the probability density function. Section 5 describes the network layer to introduce the peer-to-peer network topology simulation based on a pipe technology. Section 6 describes the storage layer to introduce the storage optimization mechanism based on shared storage and sequential reading and writing. Section 7 describes the contract layer to introduce the smart contract parallel replay technology.

## 2. Related Work

The ideas of the simulation of blockchain systems can be divided into two categories: direct simulation and model simulation. Direct simulation refers to running all of the operating steps of a blockchain system, which naturally has authenticity and scalability. However, because a large number of nodes occupy system resources, such as computing and storage resources, the efficiency of this method is low. Model simulation refers to establishing the event model of blockchain operation and calculating the simulation results according to the event model. This method is naturally efficient. However, since a blockchain protocol is not really running, the simulation’s effect cannot be guaranteed. Moreover, new blockchain protocols need new simulation models to be established, resulting in poor scalability.

At present, there are some simulation tools for simulating blockchain systems. These simulators generally serve specific experimental objectives and are only applicable to specific scenarios. They will be analyzed in the following.

Pongnumkul, S. et al. [17] built a single-node blockchain simulation environment based on the direct simulation method. Its design goal was to test the impacts of different numbers of transactions on the performance of a blockchain platform. Since only a single node existed in the implemented simulation environment, this meant that the simulation environment could not analyze the impact of the underlying peer-to-peer network on the blockchain data transmission process, nor could it judge the role of the consensus mechanism in the process of new block generation.

BLOCKBENCH [18] is a blockchain performance test platform based on direct simulation. It can simulate some important components of blockchain systems, such as consensus mechanisms and smart contracts. By using the direct simulation method, the authenticity of the platform is effectively guaranteed. It can perform small-scale simulations on different types of blockchain systems well. However, the clients of various blockchain systems run on a single local node by default. They are not optimized for the simultaneous operation of a large number of local nodes, making it difficult for this simulation tool to conduct large-scale network simulations.

VIBES [16] is a model-based simulation tool. It mainly models the network layer of a blockchain system and can conduct simulation experiments related to the network layer. This simulation tool can simulate large-scale blockchain networks and can be used to analyze the evolution process of the underlying peer-to-peer network topology of a blockchain system. However, the communication process between the internal simulation nodes is modeled as an abstract behavior. Simulation nodes do not run real blockchain network protocols, which means that it is impossible to verify the internal security risks of existing blockchain network protocols.

SimBlock [19] and BlockSim [20] are simulation tools that are implemented by using event models. Both establish an event model in a blockchain system, which is based on the generation of new events to drive the simulation environment to run. SimBlock models the block generation and network propagation processes; in particular, it analyzes the impact of the routing algorithm of a blockchain node and the network relay structure on the block propagation process. BlockSim implements an incentive layer, connector layer, and system layer; it especially models the consensus mechanism, and it can simulate the state transition process brought about by the generation of new blocks. Both of these can effectively simulate a blockchain system within the scope of the design goal, but neither of them supports manual transaction initiation in the simulation process, nor the simulation of smart contracts. The correctness of the simulation results also depends on the assumptions of the parties involved in the network—for example, miners could include transactions that offer the highest fees. The simulation of other special cases that do not meet the assumptions cannot be realized.

In general, the direct simulation method has bottlenecks in consensus computing, network management, data storage, and contract execution. The main problems of the model simulation method are the lack of blockchain functions, limited application fields, weak scalability, and need for verification of the authenticity.

In order to facilitate the analysis of different simulators, we divide the functions supported by simulators into two categories: parameters and metrics. Parameters refer to the configuration options that can be changed, and metrics refer to the statistical data obtained with simulation experiments. The parameters and metrics supported by the simulation platforms are shown in Table 1. The parameters and metrics in the table are classified according to the network layer, storage layer, consensus layer, and contract layer, where P represents parameters and M represents metrics.

Different simulators support different parameters and metrics. The results of comparing the parameters and metrics listed in Table 1 with other simulators are shown in Table 2 and Table 3.

## 3. System Architecture

This section introduces the overall operation logic and level division of the blockchain system simulation platform. This section first explains the logical relationships among the simulation network, simulation node, and blockchain protocol in the simulation environment, and it then introduces the overall running process of the simulation environment. Finally, it introduces the design objectives and specific connotations of the consensus layer, network layer, storage layer, and contract layer from the perspective of implementation.

### 3.1. Overall Operation Logic

**Figure 1:**
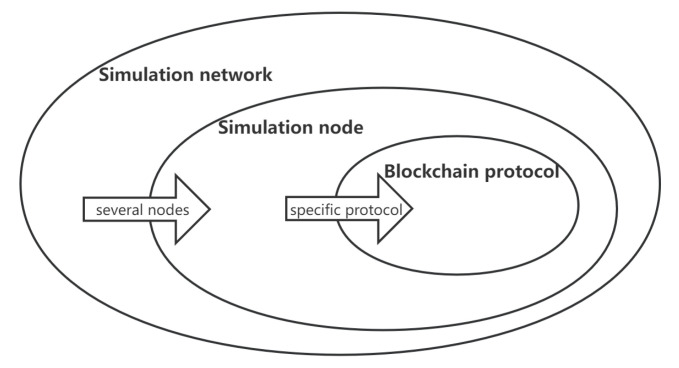
Logical relations among the components in the simulation system.

On the logical level, the blockchain simulation platform is implemented based on a three-tier architecture. The logical relationships among the simulation network, simulation node, and blockchain protocol are shown in Figure 1. There are several simulation nodes in a simulation network, and each simulation node specifies a specific blockchain protocol when it is generated. After starting the simulation node, the protocol is executed cyclically according to the predefined rules.

Creating a simulation network consists of three steps. The first step is to create a simulation network and specify the type of simulated blockchain. The second step is to specify the genesis block’s information and generate simulation nodes. The third step is to establish connections between all nodes and operate each node through the RPC interface.

**Figure 2:**
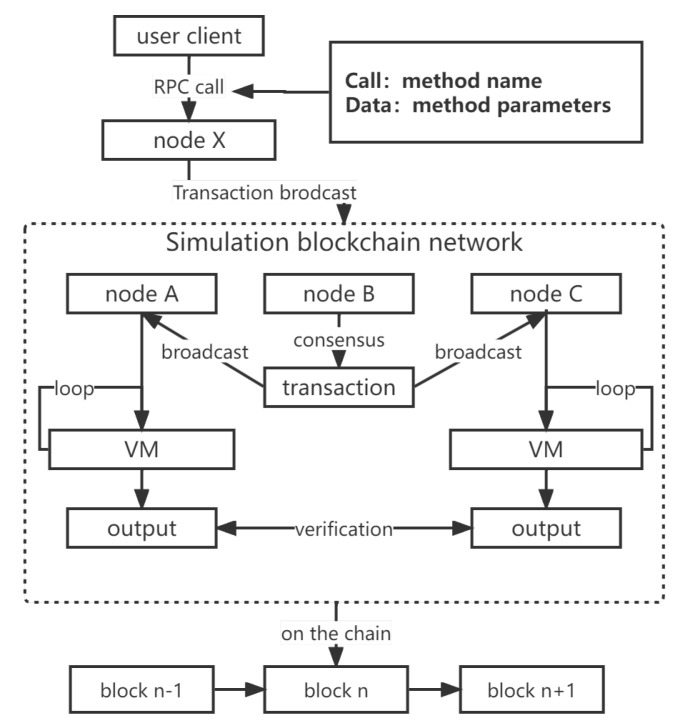
Flowchart of the blockchain simulation system’s overall operation.

The running process of the simulation network is shown in Figure 2. Each simulation node runs independently, executes its own consensus algorithm, and exposes the RPC interface to the outside. Each node in the simulation network maintains the blockchain’s content independently, runs the consensus algorithm independently, communicates with the others based on the simulation network links, and accurately reproduces the operation process of a real blockchain system. External users can initiate transactions or call smart contracts through the RPC interface provided by the simulation node. According to the operation type, the simulation node can directly return results or broadcast data to the internal simulation network.

**Figure 3:**
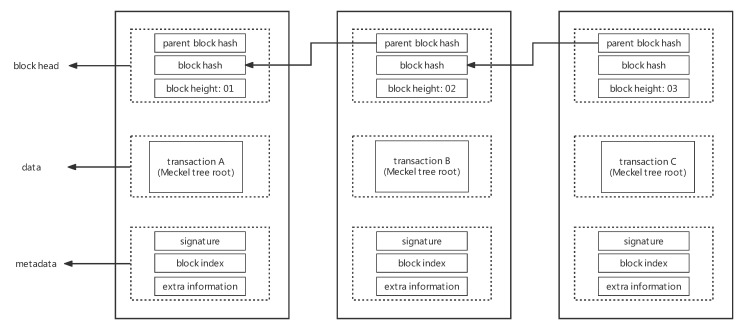
Diagram of the block structure of a simulated blockchain.

From a lower-level perspective, the blockchain simulation system is a finite-state automaton corresponding to each block one by one [21]. The block structure generated by the blockchain simulation system is shown in Figure 3. Each simulation block mainly includes two parts: the block header and block body. The block body includes two parts: ordinary data and metadata. The block header mainly records the height of the current block, the hash of the current block, and the hash of the parent block, which are used to establish the basic chain structure. Metadata record the markup information required by the consensus algorithm. Finally, the data part uses a Merkel tree to save all transaction data.

### 3.2. Layered Design Idea

**Figure 4:**
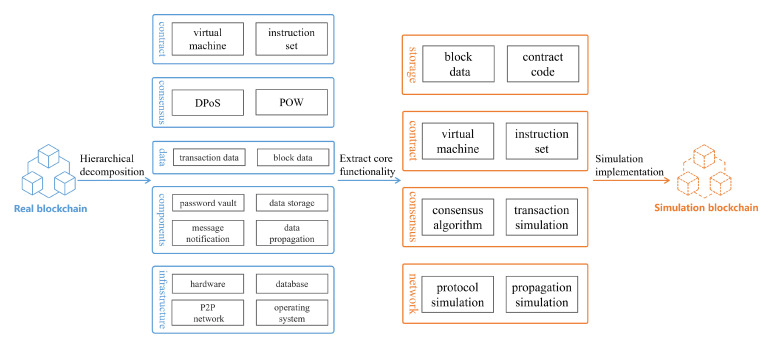
Typical hierarchical division in the blockchain simulation system.

Through a functional analysis and module decomposition of various real blockchain systems, we were able to deduce commonalities, such as the contracts, consensus, basic components, and infrastructure. These commonalities can be grouped into four layers: the consensus layer, network layer, storage layer, and contract layer. The consensus layer is the core component of various blockchain systems, and it refers to various common consensus mechanisms, such as proof-of-work mechanisms and proof-of-stake mechanisms. The network layer includes a peer-to-peer network composed of all nodes and the communication protocol used between nodes to achieve the reliable synchronization of blockchain data. The storage layer is the underlying support. It encapsulates various blockchain-specific data models and combines data integrity verification mechanisms to ensure data security. The contract layer refers to a complete set of operating environments for smart contracts, which expand the application field of the blockchain. The core of this layer is the smart contract virtual machine. The specific contents of the four layers are shown in Figure 4.

Through a study of the features of all kinds of real blockchain systems, we obtained the common content of all blockchain systems. Based on that common content, we abstracted the consensus layer, network layer, storage layer, and contract layer. The specific connotations and design objectives of these four layers are as follows.

1. Consensus layer: The confirmation of each transaction in the blockchain needs to be supported by a consensus mechanism, and consensus algorithms are the key to ensuring the decentralized nature of a blockchain. For most consensus algorithms, direct execution can be used in simulations. A proof-of-work mechanism used in a direct simulation will cause great resource consumption; thus, it is not suitable for use in a simulated environment. Due to the extensive use of proof-of-work mechanisms, consensus layer simulations must be able to simulate them on the premise of achieving the unity of efficiency and authenticity.
2. Network layer: On the whole, any blockchain can be abstracted as network communication between distributed nodes from an underlying level. The network layer needs to include simulation of the protocol at the simulation node level and the simulation of propagation at the simulation network level. In order to meet the design objectives of a network layer simulation, it needs to start from two points: simulation network protocols and simulation communication links.
3. Storage layer: All of the generated blockchain data are eventually collected into the storage layer. The characteristics of blockchain require all nodes to store a complete copy of the data separately. Only in this way can the data be guaranteed against tampering on the basis of allowing all simulation nodes to read and write blockchain data normally. The simulation of the storage layer needs to improve the overall system’s reading and writing performance as much as possible and reduce the disk space occupied by data generation in the simulation process.
4. Contract layer: The environment supporting the operation of smart contracts consists of the contract layer, which mainly includes a virtual machine and virtual machine instruction set. Simulation of the contract layer needs to provide an operating environment that is exactly consistent with the real scene for the operation of smart contracts. On the premise of realizing real and reliable simulation results, the simulation of the contract layer also needs to meet the actual requirements of the simulation system, including the fast replay of historical block data and fast creation of new nodes.

As long as the same running results can be obtained from the perspective of an external observer, the simulation can be considered complete. Therefore, it only needs to realize all of the functions of these four levels and connect the simulation nodes in a specific way to achieve the simulation.

## 4. Proof-of-Work Simulation Technology Based on the Probability Density Function

This section introduces the simulation method for the consensus layer and explains the reason for why the proof-of-work mechanism cannot be directly simulated. A proof-of-work simulation technology based on the probability density function is proposed, and the authenticity of this method is verified with theoretical and real scenarios.

### 4.1. Simulation Design Objective and Expected Effect of the Consensus Layer

In the simulation work of all four levels, the simulation of the consensus algorithm is the core content of the whole work of simulation. From the level of the simulation nodes, the existing consensus algorithms can be classified according to the selection method for the block generation node. This method can divide consensus algorithms into four categories: competition, election, random, and others. The execution processes for the above four consensus algorithms can be divided into three types of processes: generating blocks, verifying blocks, and submitting blocks. In a simulation environment, it is only necessary to implement the above three processes to realize the direct simulation of various consensus algorithms. In other words, a consensus algorithm implements three operations: generation, verification, and sealing, and these can be supported in a simulation environment. However, for proof-of-work algorithms in the competition category, the direct simulation method will cause all simulation nodes to repeatedly perform hash computations, which will cause a great waste of resources. Therefore, it is necessary to carry out specific research on the simulation of proof-of-work mechanisms to avoid these problems.

Most of the mainstream public chain systems that are currently in operation are based on a proof-of-work mechanism, such as SHA256 [22], which is used by Bitcoin, and Ethash [21], which is used by Ethereum. For proof-of-work algorithms such as SHA256 and Ethash, the simulation environment needs to test the security of the blockchain network under arbitrary hash rate scenarios. Specifically, it needs to be able to dynamically adjust the hash rate of a node and simulate the required block generation time under any hash rate level.

However, in the simulation environment, it is impossible to run all kinds of proof-of-work algorithms because of the limitation of single-machine performance and the simulation requirements of a large number of nodes. In this section, we will prove that all proof-of-work consensus algorithms conform to a probability density function with a normal distribution. Therefore, based on the probabilistic characteristics of the proof-of-work mechanism and the characteristics of the simulation environment, this paper explores a general algorithm that is suitable for the simulation environment of a blockchain system. This algorithm derives the probability density function of each node’s block generation according to the corresponding hash rate of each node and a difficulty calculation function designed by the blockchain system.

### 4.2. Mathematical Principle and Algorithm Implementation

The process by which a single node computes a hash value and determines whether the block difficulty is met can be regarded as a discrete random variable. Assuming that the output results of the current hash algorithm are uniformly distributed, the probability of blocks in each computation process is equal, so the computation process for a single node satisfies the binomial distribution. We treat a calculation process carried out n times as a random variable $X_{1},X_{2},\ldots ,X_{n}$, which is independent and identically distributed and has finite mathematical expectation and variance: $E\left(X_{i}\right)=\mu ,D\left(x_{i}\right)=\delta^{2}\left(i=1,2\ldots \right)$. Then, its distribution function satisfies the mathematical expectation in Equation (1):

(1) $$F_{n}\left(x\right)=P\left\{\frac{\sum_{n}^{i=1}X_{i}-n\mu }{\delta \sqrt{n}}\leq x\right\}$$

where *n* represents the total number of calculations, *x* represents the number of times needed to meet the difficulty requirements, μ represents the mathematical expectation, and δ represents the variance.

Because of the huge number of calculations, the above formula satisfies Equation (2) according to the central limit theorem:

(2) $$\begin{array}{c} \lim_{n\to \infty }F_{n}\left(x\right)=\lim_{n\to \infty }P\left\{\frac{\sum_{i=1}^{n}X_{i}-n\mu }{\sqrt{n}\delta }\leq x\right\}=\frac{1}{\sqrt{2}\pi }\int_{-\infty }^{x}e^{-\frac{t^{2}}{2}}dt \end{array}$$

Equation (3) states that when *n* is large, the random variable $Y_{n}$ approximately follows the standard normal distribution *n*(0,1). Therefore, Equation (4) indicates that the block behavior approximately follows the normal distribution $N(n\mu ,n\delta^{2})$. Let the block difficulty be D, let the node hash rate be C, and let the block generation time be T; then, we have $\mu =\frac{1}{d},\delta^{2}=\frac{1}{d(1-\frac{1}{d})},n=ct$. Finally, the block generation behavior and block generation time of a single node meet the normal distribution $N(\frac{ct}{d},\frac{cd-1)}{d^{2}})$. Using this conclusion, the distribution of the number of blocks out of each node in a fixed time can be calculated.

(3) $$Y_{n}=\frac{\sum_{i=1}^{n}X_{i}-n\mu }{\sqrt{n}\delta }$$

(4) $$\sum_{i=1}^{n}X_{i}=\sqrt{n}\delta Y_{n}+n\mu$$

In particular, the number of computations required for the generation of the next block can be calculated by using a Pascal distribution. Equation (5) is a Pascal distribution formula, and the in the simulation environment, *k* = 1 and $p=\frac{1}{d}$. Based on the above parameters, the probability density function of the number of block generations required by the simulation node is obtained, and the calculation times can be obtained by sampling from it. Then, the calculation times are divided by the simulated hash rate of the node to obtain the simulation block generation time.

(5) f(k;r,p)≡Pr(X=k)=k+r−1r−1(1−p)kpr

In summary, as shown in Algorithm 1, the block generation time of a single node can be calculated by using the block difficulty, the simulated hash rate of the node, and a probability density function with a Pascal distribution.   
**Algorithm 1:** Algorithm for simulating the roof-of-work mechanism.
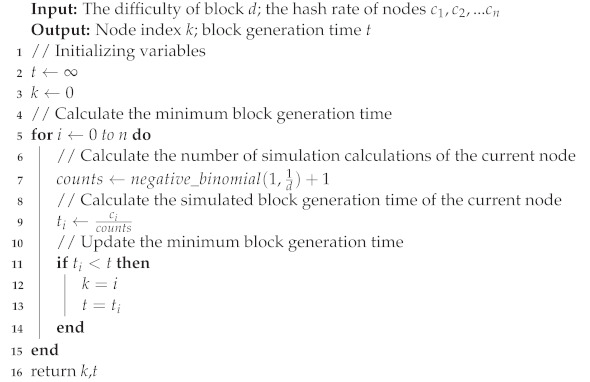


### 4.3. The Validation of the Simulation Results of the Proof-of-Work Algorithm

**Figure 5:**
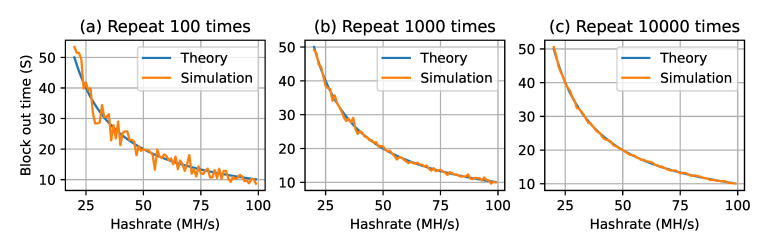
The simulated block generation time and a comparison with the theoretical value.

In order to verify if the calculation result of the algorithm matches the theoretical value, an experiment was designed to calculate the difference between the simulated block generation time and the theoretical value. In the experiment, the difficulty of the fixed block was $10^{9}$, and the final result is shown in Figure 5. The two curves in this figure represent the theoretical block generation time and the simulated block generation time. Subfigures (a), (b) and (c) show the results of 100, 1000 and 10,000 calculation times. The experimental results show that the average value of the simulated block generation time gradually approached the theoretical value with the increase in the number of experimental repetitions.

In order to verify the degree of fit between the above simulation methods and the real environment, the hash rate level and average daily block generation time of the Bitcoin network and Ethereum network in the last year were obtained, and simulation experiments were conducted for the SHA256 and Ethash proof-of-work algorithms.

**Figure 6:**
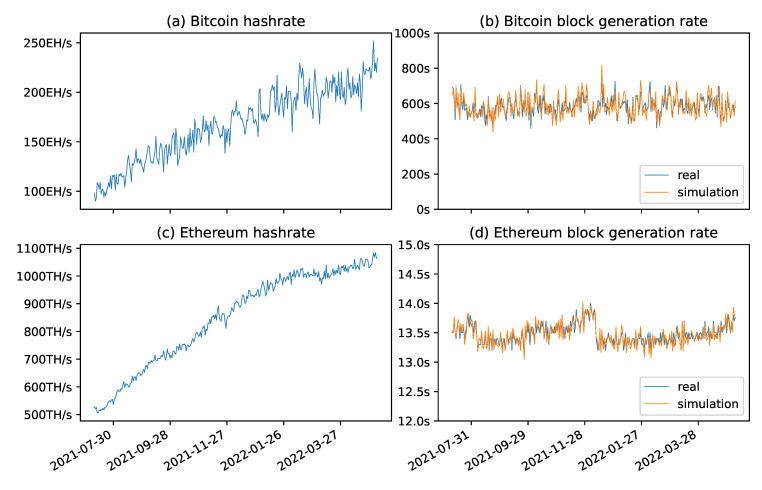
Comparison of the block generation times.

In the two simulation experiments, the daily hash rate level was used as the input parameter to calculate the block generation time, and the calculated results were compared with the block generation time in a real environment. The final experimental results are shown in Figure 6. subfigures (a) and (c) on the left show the hash rate of the whole of the Bitcoin network and the Ethereum network in the last year, and subfigures (b) and (d) on the right, respectively, draw the real daily block generation time and the simulated block generation time of the corresponding network. By comparing the changes displayed by the data on the two broken lines, it is not difficult to find that the trend of the block generation time in the simulation environment was basically consistent with that in the real environment, and the variance of the block generation time in the simulation conformed to the theoretical expectation.

Based on the above experimental results, it is concluded that the current probabilistic algorithm can simulate the running state of a blockchain network under any difficulty and any hash rate, and it can be used to realize the high-fidelity simulation of the block generation time of the proof-of-work algorithm.

## 5. Peer-to-Peer Network Topology Simulation Based on a Pipe Technology

This section studies the simulation method for the network layer. In order to realize the simulation of the underlying peer-to-peer network, this section is divided into two parts: the network protocol simulation and communication link simulation. First, the network protocols of various blockchain systems with different network types are summarized in two steps: node discovery and data communication. Then, the pipe technology is used to simulate the underlying physical links.

### 5.1. Design Objective and Expected Effect of the Network Layer Simulation

The design goal of the network layer simulation was to realize various blockchain network layer protocols, select efficient simulated communication methods, and, finally, realize a real and efficient simulation network.

Any blockchain can be abstracted as network communication between distributed nodes from the bottom layer. From the perspective of an external observer, as long as the same network traffic data can be obtained, a simulation can be considered complete. The bottom layer of blockchain technology depends on the establishment of a peer-to-peer network. One of the goals of the simulation environment is to realize the simulation of a peer-to-peer network architecture, so it is necessary to realize a simulation framework of a peer-to-peer network layer that is suitable for a blockchain system.

The design goal of the network layer simulation framework is to standardize the communication between blockchain nodes and the network simulation framework without repeatedly developing similar underlying simulation frameworks for various blockchain systems. Based on the network layer framework, a variety of security analysis tools can be quickly integrated, and a user-customized blockchain system can be implemented with minimal changes.

### 5.2. Specific Content of the Network Layer

This section will introduce the technical scheme of the network layer simulation from four perspectives: the communication protocol, coding scheme, link simulation, and a comparative experiment. The following will first introduce the communication protocols that need to be run in the network layer of a typical blockchain system, then introduce the encoding scheme for communication data selection in the simulation environment, explain the unique advantages of the pipe technology as the underlying communication link, and, finally, verify the final effect through experiments.

#### 5.2.1. Working Principles of the Peer-to-Peer Network

**Figure 7:**
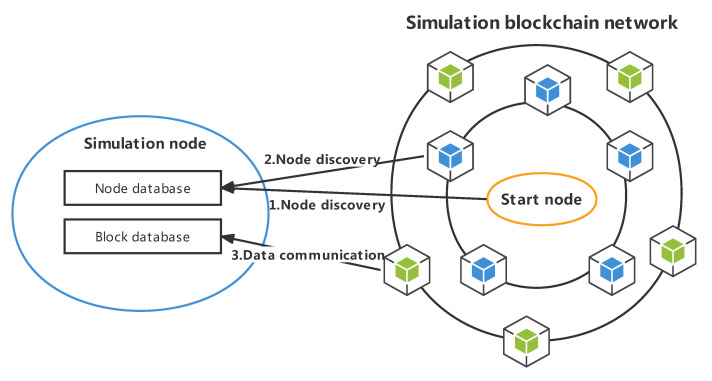
A node’s interaction with a peer-to-peer network.

Each node in a blockchain system can operate normally and participate in the whole network depending on the efficient operation of the underlying peer-to-peer network. The process of initializing a node to join a peer-to-peer network can be divided into two processes—node discovery and data communication—as shown in Figure 7.

Specifically, the node discovery process can be divided into three steps. First, a series of seed nodes for the local node are specified, and they are saved in the local node database. Then, the local node executes the node discovery algorithm on the seed node to obtain the node record information of other nodes. Finally, the local node saves the obtained records to the node database and uses the routing algorithm to select a specific node from the database to continue the query, thus repeating the cycle.

The process of data communication can be divided into two steps. First, a specific algorithm, such as the Kademlia algorithm, is used to select peer nodes with a short distance from the local node database. Then, encrypted handshakes and sub-protocol handshakes are carried out with peer nodes. After verification, an encrypted communication link can be successfully established for block data exchange. The following uses the Ethereum network as an example to detail the protocols involved in the node discovery and data communication processes.

There are two main protocols running on the peer-to-peer network of Ethereum, namely, a node discovery protocol based on UDP and an RLPx protocol based on TCP [23]. Local nodes need to obtain the basic information of other nodes through the node discovery protocol at the beginning, and then they establish encrypted data communication links with each other through the RLPx protocol after selecting the corresponding nodes. The node discovery protocol consists of three steps: 1. Communicating Ping and Pong packets; 2. Communicating ENRRequest and ENRResponse packets; 3. Communicating FindNode and Neighbors packets. The operation process of RLPx mainly includes three steps: 1. Encryption handshake stage; 2. Protocol handshake stage; 3. Starting and running application layer protocols.

#### 5.2.2. Node Discovery Protocol

In the node discovery protocol, nodes probe each other to discover other nodes in the network. In order to join a blockchain network and start block synchronization, an Ethereum client will designate a set of seed nodes to discover other active nodes and save their information in the corresponding bucket. In order to communicate node records between nodes as quickly as possible, the Ethereum node discovery protocol uses UDP as the transport layer protocol. There are six message types in the v4 version of the protocol: Ping, Pong, FindNode, Neighbors, ENRRequest, and ENRResponse. The Ping and Pong packets are used to check if a remote node is alive. The Neighbors packet returns 16 entries to the nearest node in the FindNode packet each time.

**Figure 8:**
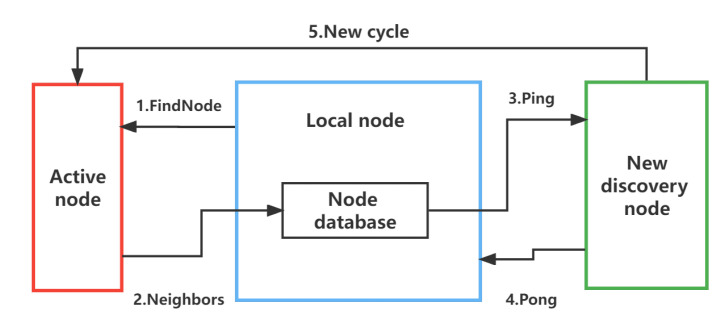
Sending and receiving processes of the node discovery protocol packets.

The process of running the node discovery protocol and adding node records to the node database is shown in Figure 8. Within the first RTT, the FindNode packet is sent locally, the Neighbors packet returned by the remote node is received, and the 16 node records saved in the packet are saved to the node database. During the second RTT, the local node sends a Ping packet to the remote node to try to activate the remote node. After receiving a Pong packet, it indicates that the peer party is alive. After that, the above operations are repeated for the remaining nodes.

#### 5.2.3. Node Security Communication Protocol

The next step after node discovery is to exchange data with the newly discovered node. This process needs to implement a reliable transmission protocol that can achieve encrypted communication, and a typical one is the RLPx protocol. The RLPx protocol is used to establish a secure TCP connection between two nodes. The process of establishing a connection can be divided into two steps: the encryption handshake stage and the subprotocol handshake stage.

1. Encryption handshake stage: Symmetric encryption keys are constructed based on ECIES for subsequent communication [24].
2. Subprotocol handshake stage: The two parties communicate with each other about the name and version of the subprotocol and select an appropriate protocol for data transmission. The two communication parties first send a HELLO message to each other, which contains their node ID, DEVp2p protocol version, client name, supported application layer protocol, and local listening port number (default 30303) [25].

### 5.3. Node Life Cycle and Pipe Technology

In traditional simulation technologies, in order to simulate an environment similar to that of real blockchain node communication, it is necessary to use the local loopback address and occupy the local port to realize the simulation of a peer-to-peer network [26]. As a result, the network communication between nodes depends on the network interface of the operating system, and the number of node simulations is limited by the locally available ports.

In the simulation platform, various types of blockchain systems will share the same peer-to-peer network layer design. According to the characteristics of the simulation environment, we explore the use of a pipe technology to directly simulate the synchronous and full-duplex network connections in memory to avoid the limitations of traditional local socket communication.

**Figure 9:**
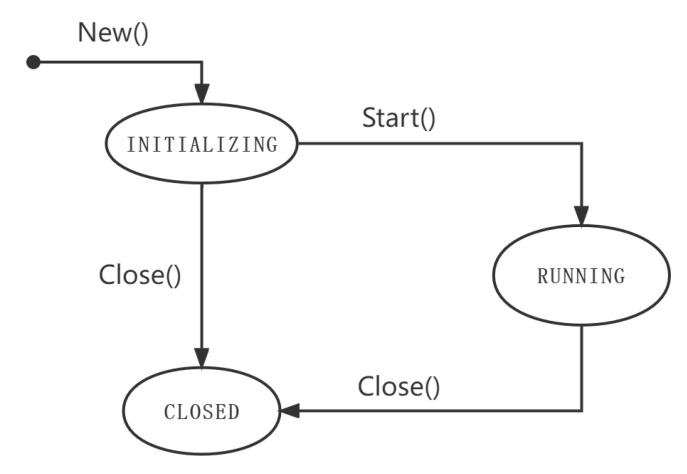
State transition relationships of nodes in the simulation network.

A node in the simulation network is a collection that manages a set of system resources and registers network layer services. The life cycle of the simulation node includes three states: INITIALIZING, RUNNING, and CLOSED. The relationships between the three states are shown in Figure 9.

INITIALIZING: Creating a node in the initial state involves allocating the required resources and registering the network protocol. The resources allocated by each node include the data folder on which the node is allowed to operate, the RPC service, which is registered externally, and the key-value database, which can be read and written. Once the resource allocation is complete, the next step is to define the network layer protocols that the new node can run, such as the node discovery protocol and data communication protocol that were described above.

RUNNING: After resources are allocated and network protocols are registered, the new node is ready and can be started at any time. After the node is started, the node continues to implement the specified network layer protocol until the protocol runs successfully. A running node cannot register any network layer protocols.

CLOSED: A node in the closed state will release all resources that it occupies. The release of resources depends on the status of the node before it is shut down. When a node in the initial state is shut down, all allocated system resources are released. When a node in the running state is shut down, in addition to releasing system resources, all network connections and running RPC services are shut down.

The implementation of the network layer protocol is based on four core operations: Connect, Send, Read, and Close. In any blockchain system, the network layer protocol can be implemented with the above four methods, so it can be used as the underlying communication link by simulating the above operations through the memory pipe.

According to the prototype system, this technology can be used to simulate any number of nodes within the allowable memory range, and it prevents losses caused by unnecessary packet packaging in the network layer and transport layer. In addition, using a pipe to simulate network communication avoids the influence of the operating system on network packet sending, and the simulation of flow control, packet loss rate, and delay jitter of connections between nodes can be more accurately realized.

### 5.4. Communication Performance Comparison Test of the Simulation Environment

**Figure 10:**
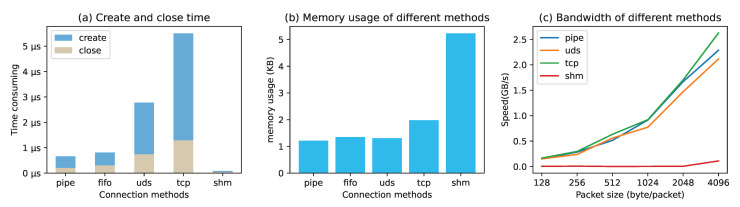
Performance comparison of various simulation link implementation methods.

In order to verify the actual effects of different simulation links, the communication performance of different underlying links was compared and experimentally tested; these links included a memory pipe (PIPE), a named pipe (FIFO), a Unix domain socket (UDS), a network socket (TCP), and shared memory (SHM). This experiment tested the average time taken for connection creation and disconnection, the memory usage of a single link, and the upper limit of the communication bandwidth for the five methods described above, and the final results are shown in Figure 10. It can be seen in subfigure (a) that the Unix domain socket scheme and TCP scheme took a long time to create and close connections. It can be seen in subfigure (b) that the shared memory scheme had a slightly higher memory consumption. It can be seen from subfigure (c) that, except for the shared memory scheme, there was little difference in the upper bandwidth limits of various schemes. The experiment was run in the environment of an Intel(R) Xeon(R) Gold 5220 CPU at 2.20 GHz *72 CPU with 128 G RAM and 2 TB SSD, and 100 nodes were configured to establish a complete graph network.

The Unix domain socket scheme requires the creation of local IPC files for each node, so it can take a long time to create connections. Local TCP communication requires frequent requests for and release of local ports. As a result, it takes much longer to create and destroy connections than with other schemes. In addition, the number of connections is limited due to the port numbers. Although the shared memory method takes a very short time to create and close because it does not need to establish a connection, it is not advantageous in terms of memory occupation and communication bandwidth. In addition, shared memory requires a fixed memory area for each link, so it occupies a large amount of memory. Moreover, an additional locking mechanism may be used to significantly reduce the bandwidth and increase the implementation complexity in order to achieve synchronous communication between the two sides.

After a comprehensive analysis of the above experimental results, it is not difficult to find that the memory pipe scheme has obvious advantages in terms of connection creation efficiency and memory space occupation, and it does not have obvious disadvantages in communication bandwidth. Therefore, it can be concluded that using the memory pipe technology as the underlying link support for the peer-to-peer network can meet the needs of the simulation environment.

## 6. Storage Optimization Mechanism Based on Shared Storage and Sequential Reading and Writing

To improve the query performance and solve the problem of redundant data storage, this section focuses on the simulation of the storage layer. In order to improve the query performance, this section first introduces the underlying storage format of a flat dataset. To solve the problem of redundant data storage in the simulation environment, a shared storage scheme is proposed.

### 6.1. Design Objective and Expected Effect of the Storage Layer Simulation

In the consensus stage of the simulation environment, after a block is successfully connected to the chain, the blocks that have reached consensus on the blockchain all contain the same data, and it will cause great waste for all simulation nodes to repeatedly store this part of the data. In addition, LevelDB’s performance deteriorates due to the random distribution of hash values and the growth of the data. As a result, the disk reading and writing performance continuously deteriorates. Storage optimization can be divided into two directions: reducing storage overhead and improving reading and writing performance. Therefore, in order to solve the above two problems, this paper proposes a storage optimization mechanism based on shared storage and sequential reading and writing.

In this method, a shared storage mechanism of nodes is used to uniformly save the confirmed data to reduce the occupation of disk space. The essence of this method is the storage of the blockchain’s data in a hierarchical manner. The first level is an in-memory database for storing pending transactions and newly generated blocks. The second level is the LevelDB database, which holds new data. The third level is used to save the fully confirmed data, and it is a flat dataset that will not be changed. The data in the flat dataset are linear data that are stored in one dimension, and the storage location is directly located according to the block height without using a hash value for the index. Therefore, when a large number of traversal queries are executed, the sequential reading and writing performance of disks can be fully utilized to significantly improve the sequential query speed.

The simulation data are divided into shared data and new data, which greatly reduces the storage space required by the simulation environment. At the low-level database reading and writing level, all simulation nodes will use the same shared data according to the progress of the consensus algorithm in constantly carrying out the transformation of new data and shared data.

### 6.2. Flat Dataset

Data in the shared storage area are managed through a storage format called a flat dataset. The underlying storage can be divided into index files and data files according to the file types. The data file saves the data generated during the operation of the blockchain, while the index file records the offset of the real data in the data file.

The index file consists of several index entries with a length of 6 bytes. The first 6 bytes of the index file are the index offset, which is used to record the starting position of the real data in the data file. Each index entry can be used to locate the location of a piece of data. The first two bytes store the data file number, and the last four bytes store the end position of the real data in the data file. The specific formal definition is as follows:

1. <index-file> → <index-offset> <index-entry>+
2. <index-offset> → 0x00 0x00 <uint32>
3. <index-entry> → <uint16> <uint32>

Let the size of the index file be t bytes; then, the formula for calculating the total number of saved data is: $\frac{t}{6}-1$. Let the data to be queried be item K; then, the byte position in the index file is: 6k.

The data file holds only pure binary data and does not contain any other information internally. By using this design, the data of adjacent blocks are continuously stored, which can improve the efficiency when sequentially reading a large number of block data.

Querying complete data from a flat dataset consists of two processes: obtaining index information and locating the real data. Firstly, the index entry of the queried data is obtained from the index file, from which the data file number and the corresponding data file byte position are resolved. The next step is to use the index information above to read all of the real data from the data file. Algorithm 2 shows the algorithmic process of locating data in a flat dataset.    
**Algorithm 2:**Data location algorithm for a flat dataset.    **Input**: table name tab; data index *i*    **Output**: binary data res**1** // Reading index files**2** entry-index ← i*(6+1) // Calculate the byte position of the index entry**3** index-file ← open(tab +”.idx”) // Open the index file of the corresponding table**4** index-file.seek(entry-index)**5** // Parsing index entries**6** datafile-num ← index-file.read(2) // Read the data file number**7** datafile-index-begin ← index-file.read(4) // Read the start position of the real data**8** index-file.read(2) // Read the data file number**9** datafile-index-end ← index-file.read(4) // Read the end position of the real data**10** // Read real data from a data file**11** data-file ← open(tab + datafile-num + ”.dat”) // Open the data file**12** index-file.seek(datafile-index-begin)**13** res ← data-file.read(datafile-index-end - datafile-index-begin) // Read result**14** return res

### 6.3. Verification of the Storage Optimization Effect

**Figure 11:**
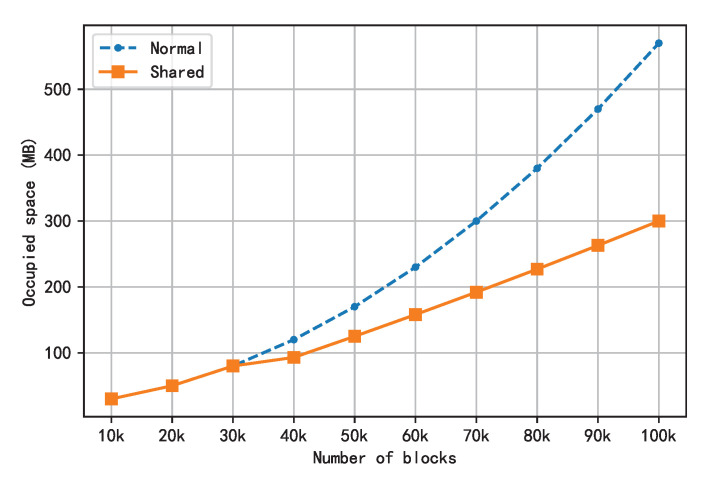
Comparison of the space occupation between shared storage and common storage.

In order to verify the optimization effect of the shared storage scheme on space occupation, 10 nodes were created in the simulation environment, and the contents of the first 100,000 blocks of Ethereum were replayed. Figure 11 shows the disk space usage before and after the optimization when the threshold was set to 30,000.

As can be seen from the data in Figure 11, since the first 30,000 blocks did not reach the threshold, LevelDB was completely used as the underlying storage in both cases, with no difference in the space occupied. When the threshold was exceeded, the two curves bifurcated. With the increase in the block height, the content of the block was moved to the shared storage, and the space occupation gap between the two curves gradually widened.

**Figure 12:**
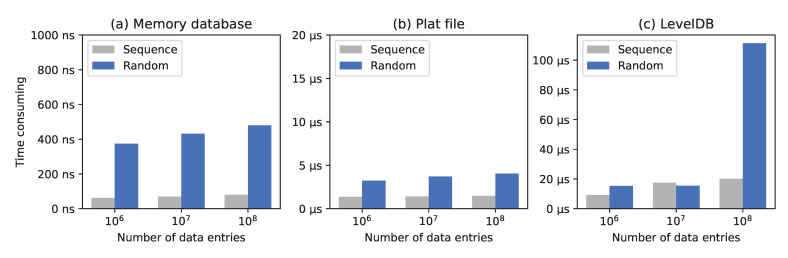
Comparison of the reading times of different data storage schemes.

In order to verify the query performance of the flat dataset, the average query times of the in-memory database, flat dataset, and LevelDB with millions, tens of millions, and hundreds of millions of data items were calculated. In order to approach the query characteristics of a real blockchain scenario, all of the queried data were randomly generated 32-byte hash values, and the experimental results are shown in Figure 12. The figure shows the sequential and random query times for the three storage media, each of which was tested with three different orders of magnitude of data. Subfigures (a), (b) and (c) are the experimental results of memory database, plat file and LevelDB respectively.

In subfigure (a), the memory database was used as the test benchmark to calibrate the upper limit of the query performance. subfigure (c) shows that LevelDB’s random reading performance deteriorated significantly when the number of saved data entries was increased. It can be seen in subfigure (b) that the performance of sequential and random querying on the flat dataset was relatively stable, and the time consumption was significantly less than that of the LevelDB data before the performance deterioration.

Based on the above experimental results, it can be concluded that the shared storage scheme can effectively reduce the repeated storage of redundant data in the simulation environment without affecting the simulation’s effect. The design of the flat dataset can achieve fast querying of confirmed data, thus effectively avoiding the performance degradation of LevelDB due to the increase in data entries.

## 7. Smart Contract Parallel Replay Technology

This section focuses on the contract layer simulation. From the perspective of the contract layer simulation, in order to realize the fast replay of transactions, fast import of blocks, and fast creation of nodes, this section proposes smart contract parallel replay technology. This technology solves the problem of the waste of computing resources caused by the repeated replay of historical blocks, and it can also make full use of the processor performance to speed up the block replay.

### 7.1. Design Objective and Expected Effect of the Contract Layer Simulation

The design goal of the contract layer simulation is to realize the rapid replay of massive transactions and, finally, achieve the fast creation of new nodes and fast import of historical blocks.

With the continuous operation of the simulation network, the block height continuously increases, and every new node needs to synchronize all of the historical blocks. When importing the original simulation network, all simulation nodes are required to replay all historical blocks. In both cases, the simulation nodes need to replay the calling process for massive smart contracts.

However, due to the characteristics of smart contracts, the states of the front and back blocks depend on each other. The execution of contract codes in the latter blocks depends on the execution results of the previous blocks. Therefore, the current smart contract virtual machine can only replay all historical blocks linearly and cannot take advantage of the multi-core performance of mainstream processors. At present, it takes more than 13 days to replay all blocks for the Ethereum archive mode [27].

Based on these reasons, using the existing smart contract virtual machine directly will make the time-consuming processes of new node creation and block import unacceptable. In order to solve the above problems, the smart contract state cache and parallel replay technology are proposed for the contract layer. The experimental results show that the use of this technology can significantly reduce the new node creation time and greatly speed up the import of historical block records.

### 7.2. Smart Contract State Cache Implementation Scheme

In the process of creating a new node, all of the historical blocks that need to be replayed by the new node have been verified by the execution of the rest of the simulation nodes. Continuing to repeatedly execute the contract code on the new node will not improve the authenticity of the simulation system. Therefore, the execution state of the smart contract can be cached in the simulation environment, and the new node can be quickly restored to the cache position when it is created so as to solve the problem of the new node creation process taking a long time.

In addition to the creation of new nodes, the smart contract running state is also cached in order to implement the parallel replay of the smart contract. Due to the dependency between the previous and subsequent blocks on the blockchain, each execution of the contract code will cause a change in the node’s world state. Each execution of the contract code may cause changes in the account balance, changes in the status of other contracts, and changes in contract variables. These changes in the state of the world occur sequentially as the height of the block increases. In order to achieve the parallel replay of the contract code, it is necessary to eliminate the inter-block dependencies. One solution is to cache the on-chain smart contract state of each block.

The essence of caching smart contract execution results is saving the real-time states of all nodes in the network. Each node can be regarded as a finite-state automaton. Each block generated is equivalent to executing the automaton’s state transition function [21]. The real-time state of each node can be represented by a world state tree. By solidifying the world state tree of the current position of the node, a smart contract state cache with a specified block height can be obtained. To build a smart contract state cache, the transaction data stored in all historical blocks should be replayed sequentially. Then, when the height of the block to be cached is reached, the world state of the blockchain is obtained. Finally, the cache data are saved to LevelDB through the storage layer.

### 7.3. Smart Contract Parallel Replay Implementation Scheme

The smart contract state cache solves part of the problem of replaying the contract code by waiting until the contract code has been replayed at the remaining simulation nodes. To improve the overall execution efficiency of smart contracts in the simulation environment, it is fundamentally necessary to implement the parallel replay technology for the contract code and make full use of the multi-core performance of current processors.

The smart contract parallel replay process first reads the state cache of the specified block height from LevelDB, and then starts the contract code replay from multiple starting points at the same time. The detailed execution process is as follows:

1. First, the initial configuration of parameters is set, including the start block, end block, block packet size, and upper limit of parallel processing.
2. The blocks within the starting and ending range are divided into several block groups according to the block packet size, and these block groups are used as the execution units of parallel processing tasks.
3. The saved smart contract state cache is read from LevelDB, and then the block contents corresponding to the block height are read. A smart contract virtual machine environment is created, and each transaction from this block is replayed.
4. When the replay of a block group is completed, the state of the end position is saved for final verification.
5. Steps 3 and 4 are repeated until the number of concurrent threads reaches the upper limit for parallel processing or all blocks have been replayed.

Algorithm 3 shows the execution process for parallel replays. The execution process of the entire algorithm is divided into two parts, which are replay and verification. First, the block data within the required range are replayed in parallel, and then it is verified if the replay state is consistent with the saved state.    
**Algorithm 3:** Block parallel replay algorithm.
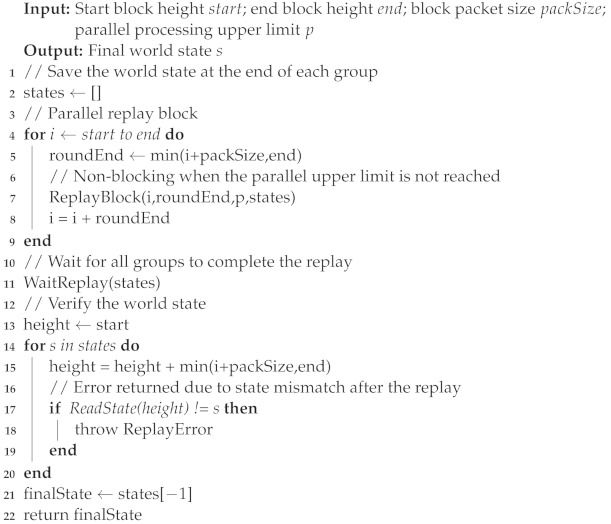


### 7.4. Efficiency Comparison Test for the Parallel Replay

In order to verify the optimization effect of the parallel replay, the first 1,000,000 blocks of the main Ethereum network were collected for a replay test. During the experiment, the performance of a single thread and of parallel numbers of 2, 4, and 8 was assessed. The experiment was conducted in the environment of an Intel(R) Xeon(R) Gold 5220 CPU at 2.20 GHz *72 CPU with 128G RAM and 2TB SSD.

**Figure 13:**
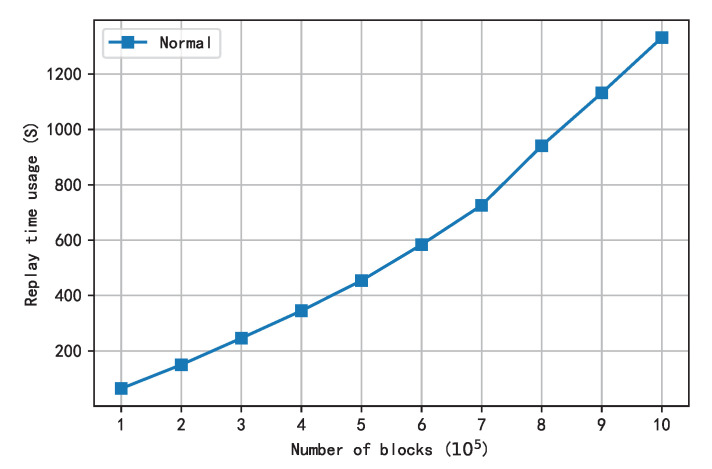
The time taken to replay the first million blocks in a single thread.

Figure 13 shows the time curve for replaying the first 1,000,000 blocks of Ethereum by using a normal smart contract virtual machine in a single thread. In the first half of the graph, the slope of the curve is relatively fixed because there are fewer block transactions on the main network. In the second half of the figure, as the block height increases, the number of transactions in each block increases, so the playback time per block increases.

**Figure 14:**
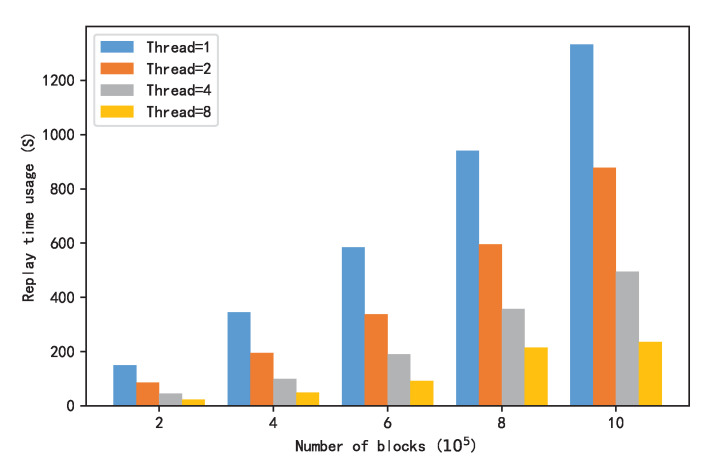
Comparison of the playback times for different threads.

Figure 14 shows a comparison of the time taken to replay the specified number of blocks while using different parallel numbers. It can be seen in the figure that with the increase in the number of parallels, the playback time of the specified block data decreased significantly.

In the case of a fixed number of parallels, the time taken to complete the replay depended on the packet that took the longest time. In Figure 14, when replaying 1,000,000 blocks, the time consumption of parallel number 2 was obviously more than half of that of a single thread. This was due to the increased volume of transactions in the second half of the block; the overall replay completion time depends on the second half of the replay time.

The above experimental results show that the efficiency of replaying a large number of blocks can be effectively improved by using the smart contract state cache and parallel replay technology and by setting relevant parameters reasonably. Based on the above methods, the simulation environment can dynamically create new nodes, and the time consumption of the history state import process is significantly reduced.

## 8. Conclusions

With the widespread application of blockchain technology, various security issues are also becoming increasingly prominent. However, due to the complexity of the blockchain system and the particularities of some security issues, the cost of the deployment of blockchain systems in real environments is high. In addition, the related security analysis and verification also have a certain destructiveness. In response to the above issues, this paper proposes the design and implementation method of a blockchain system simulation platform.

This paper studies a layered design method for the simulation of typical blockchain systems. On the logical level, the blockchain system simulation platform is implemented based on a three-layer architecture of a simulation network, simulation node, and blockchain protocol. At the realization level, this paper divides the main existing components of a blockchain into four layers, namely, the consensus layer, network layer, storage layer, and contract layer, through induction and decomposition.

Aiming at these four layers, the paper proposes a proof-of-work simulation technology based on a probability density function, a peer-to-peer network topology simulation based on a pipe technology, a storage optimization mechanism based on shared storage and sequential reading and writing, and a smart contract parallel replay technology. By means of these techniques, problems such as the waste of computing resources, low communication efficiency, and redundant storage in the simulation environment are solved. By comprehensively including all of the above levels of content, a real and efficient blockchain simulation prototype system was finally realized to provide security support for related technological research in the field of blockchain.

In future work, we will realize the combination of different components to facilitate the analysis of the performance of a variety of different types of blockchain systems. The next step will be to enhance the applicability of the above simulation methods to more and different types of blockchain systems so that they will not be limited to the public chain domain, thus helping to simplify security research in more areas.

## Figures and Tables

**Table 1:** Parameters and metrics supported by the simulation platforms.

Layer	P/M	Definition
network layer	**(P1)**Block Delay	Average delay of block propagation on the simulation link
	**(P2)**Transaction Delay	Average delay of transaction propagation on the simulation link
	**(P3)**Link Bandwidth	Maximum transmission bandwidth of the simulation link
	**(P4)**Link Latency	Average transmission delay of the simulation link
	**(M1)**Nodes	Number of nodes running in the simulation network
	**(M2)**Connections	Total number of network connections between all nodes
	**(M3)**Block Time	Average time for block propagation in the simulation network
	**(M4)**Transaction Time	Average time for transaction propagation in the simulation network
storage layer	**(P5)**Transaction Speed	Speed of generating random transactions in simulation networks
	**(P6)**Max Transaction Size	Maximum transaction size configured in the simulation environment
	**(P7)**Max Block Size	Maximum block size configured in the simulation environment
	**(M5)**Block Size	Average block size generated by the simulation network
	**(M6)**Result Chain	Blockchain for exporting simulation results
	**(M7)**Security Integration	Simulation network operation data can be obtained for security analysis
consensus layer	**(P8)**Node Hashrate	The hash rate owned by each node in the simulation network
	**(P9)**PoW Algorithm	Whether proof-of-work mechanisms are supported in the simulation environment
	**(P10)**Other Consensus Algorithm	Whether other consensus algorithms are supported in the simulation environment
	**(P11)**Miner Reward	Calculation method for the block reward used when generating new blocks
	**(M8)**Reward Statistics	Total amount of currently generated block reward
	**(M9)**Average Block Interval	The average generation time of each new block in the simulation network
	**(M10)**Average Transaction Fee	Average transaction fees for all transactions in the simulation network
contract layer	**(P12)**Contract Validation	Whether contract validation is enabled in the current simulation network
	**(M11)**Contract Execution Time	Average execution time of a smart contract in the simulation network
	**(M12)**Throughput	Average transaction throughput in the current simulation network

**Table 2:** Comparison of the parameters of typical simulators.

Name	P1	P2	P3	P4	P5	P6	P7	P8	P9	P10	P11	P12	Total
VIBES	⬤	⬤	◯	◯	⬤	⬤	⬤	◯	⬤	◯	⬤	⬤	8
SimBlock	◯	⬤	◯	⬤	⬤	⬤	⬤	◯	⬤	◯	◯	⬤	7
BlockSim	⬤	◯	⬤	⬤	◯	⬤	◯	◯	⬤	◯	◯	◯	5
BlockBench	⬤	⬤	◯	⬤	⬤	⬤	⬤	◯	⬤	◯	◯	⬤	8

**Table 3:** Comparison of the metrics of typical simulators.

Name	M1	M2	M3	M4	M5	M6	M7	M8	M9	M10	M11	M12	Total
VIBES	⬤	◯	⬤	⬤	⬤	⬤	⬤	◯	⬤	◯	◯	⬤	8
SimBlock	⬤	⬤	⬤	◯	⬤	⬤	◯	⬤	◯	◯	◯	⬤	7
BlockSim	⬤	⬤	⬤	⬤	◯	⬤	◯	◯	⬤	⬤	◯	◯	7
BlockBench	⬤	◯	⬤	⬤	⬤	◯	◯	◯	◯	⬤	◯	⬤	6

## Data Availability

All data included in this study are available upon request by contact with the corresponding author.
